# Application of a continuous nursing model based on medical treatment combination in patients with stroke sequelae

**DOI:** 10.3389/fneur.2026.1800527

**Published:** 2026-07-24

**Authors:** Xiuling Luo, Na Wang, Lei Tian, Dongyun Zhang, Yuanhong Xu

**Affiliations:** 1 Department of Neurological Rehabilitation Center, Taihe Hospital, Hubei University of Medicine, Shiyan, Hubei, China; 2 Department of Nursing, Taihe Hospital, Hubei University of Medicine, Shiyan, Hubei, China; 3 Department of Rehabilitation Therapy, Yunyang District Chengnan Community Health Service Center, Shiyan, Hubei, China; 4 Department of Outpatient Services, Taihe Hospital, Hubei University of Medicine, Shiyan, Hubei, China

**Keywords:** continuous nursing model, effect, medical treatment combination, stroke, stroke sequelae

## Abstract

**Background:**

This study aimed to evaluate the effectiveness of a medical consortium-based continuous nursing model in patients with stroke sequelae.

**Methods:**

A total of 60 patients with stroke sequelae were randomly assigned to either the intervention group or the control group, with 30 patients in each group. Patients in the control group received routine continuous nursing, and those in the treatment group received continuous home nursing from community medical institutions. The clinical nursing effects were compared between the two groups.

**Results:**

After receiving continuous home nursing from community medical institutions, patients in the treatment group had higher scores in activities of daily living (ADL), Stroke-Specific Quality of Life Scale (SS-QOL), and nursing satisfaction, as well as a lower probability of complications, readmission rate, and medical expenses than those in the control group, and the comparison was statistically significant (*p* < 0.05).

**Conclusion:**

The medical treatment combination-based continuous nursing model can promote the recovery of patients with stroke sequelae and improve their self-care ability, nursing satisfaction, and quality of life. Additionally, this model can reduce the occurrence of complications and readmission rates, maximize the utilization of social resources, and lower medical costs. Therefore, it has high social value and is suitable for promotion in clinical practice.

## Introduction

Stroke is a major neurological disorder associated with high rates of disability and mortality (1). In China, the incidence of stroke is on the rise with the acceleration of social aging. Despite the reduction in clinical mortality with the development of modern medical technology, approximately 75% of stroke patients have disabilities or impairments in cognition, self-care ability, activities of daily living, treatment compliance, and psychology after onset, which seriously affects patients’ quality of life and increases the family and social burden (2). Rehabilitation for patients is a long-term process that requires professional medical support. However, many discharged patients with stroke sequelae face challenges such as limited community medical resources, unsound social support system (3), and family members’ lack of professional medical knowledge. As a result, they fail to get professional follow-up nursing and rehabilitation treatment, leading to repeated hospitalizations. This places a significant financial and mental strain on themselves and their families (4). Therefore, the post-discharge rehabilitation and prognosis of patients with stroke sequelae have received widespread attention. A medical treatment combination suggests that public medical institutions of different types and levels in a region are combined into a cooperative alliance or medical group as a community of shared interests and a community of shared responsibilities to realize the sharing of medical resources, the interconnection of medical information, and the homogenization of medical services (5). Since the 1970s, foreign medical treatment combinations have established a relatively mature system after years of development. Foreign continuous nursing, first proposed by the College of Nursing at the University of Pennsylvania in the 1980s, can effectively improve the prognosis of various diseases at low cost (6) and is delivered within a relatively mature medical treatment combination service system (7). In China, patients prefer large hospitals with abundant high-quality medical resources to community medical institutions, which are deficient in both resources and capacity. Establishing a combination of medical treatments is an effective way to implement tiered diagnosis and treatment (8) and an important measure to deepen medical and health system reforms, optimize the structure, and adjust the allocation of medical resources (9). As the incidence of chronic diseases increases in China, continuous nursing services have received widespread attention, and a significantly increasing number of studies have been conducted, mainly involving stroke, diabetes, hypertension, and other chronic diseases. A review of literature indicates that the majority of studies have focused on continuous nursing carried out in tertiary hospitals, mainly on specific diseases and the effect of continuous nursing. While hospital-community linkage models have been proposed previously, the majority of studies lack standardized protocols, quantified training requirements, and detailed implementation parameters.

To explore the clinical effect of a medical treatment combination-based continuous nursing model on patients with stroke sequelae, a vertical continuous nursing network system was established based on medical treatment combination. A continuous nursing group was formed by tertiary medical institutions, aiming to improve the professional knowledge and skills of medical workers through theoretical teaching, operation training, business guidance, case discussion, and other training, and to provide guidance for community medical institutions in completing stroke sequelae-related continuous nursing activities in a home environment by telephone follow-up, family visit, health education and consultation based on a network platform, and other methods. This ensured that the discharged patients with stroke sequelae had a smooth transition and had access to continuous and homogeneous health nursing services in their homes, which improved their quality of life and reduced complications. Details are reported as follows:

## Data and methods

### General data

A total of 60 patients with stroke sequelae admitted to the Neurology Department of our hospital from April 2019 to March 2020 were selected, and the study was approved by Taihe Hospital’s Ethics Committee. All patients met the clinical diagnostic criteria for stroke and gave informed consent, and those with serious dysfunction of important organs such as the heart, lung, liver, and kidney, as well as serious mental disorders, cognitive disorders, and malignant tumors, were excluded. The patients were randomized as described in the randomization and blinding section below. After exclusions and dropouts, 30 patients per group completed the study and were included in the final analysis. In the treatment group, there were 18 men and 12 women, aged 38–78 years, with an average age of 52.07 ± 10.15 years. The number of days after stroke was 1–10 days, with an average of 4.47 ± 2.56 days. The degree of disability was (3 ± 1.34) according to the modified Rankin Scale; in the control group, there were 16 men and 14 women, aged 38–78 years, with an average age of (55.07 ± 11.99) years. The number of days after stroke was 1–12 days, with an average of 4.73 ± 3.01 days, and the degree of disability was (2.97 ± 1.30) according to the modified Rankin Scale. There were 8 cases of cardiogenic embolism, 9 cases of atherosclerotic thrombosis, 7 cases of lacunar thrombosis, and 6 cases of other types in the treatment group, and 10 cases of cardiogenic embolism, 7 cases of atherosclerotic thrombosis, 8 cases of lacunar thrombosis, and 5 cases of other types in the control group. There was no significant difference between the two groups in terms of sex, age, clinical symptoms, or other general data, so the interference of other factors could be eliminated. Details are shown in Tables 1, 2.

**Table 1:** Comparison of general data between the two groups.

	Treatment group	Control group	*χ*^2^/*t* value	*p* value
Number of cases	30	30		
Sex (female/male)	18/12	16/14	0.271	0.602
Age (years)	52.07 ± 10.15	55.07 ± 11.99	1.046	0.3
Days after stroke (days)	4.47 ± 2.56	4.73 ± 3.01	0.37	0.713
Degree of disability ^a^(score)	3 ± 1.34	2.97 ± 1.30	0.098	0.922

a mRS, modified Rankin Scale.

**Table 2:** Comparison of cerebral infarction types between the two groups, *n* (%).

Group	Number of cases (*n*)	Cardiogenic embolism	Atherosclerotic thrombosis	Lacunar thrombosis	Other
Treatment group	30	8 (26.7)	9 (30.0)	7 (23.3)	6 (20.0)
Control group	30	10 (33.3)	7 (23.3)	8 (26.7)	5 (16.7)
*χ* ^2^	0.63				
*p*	0.89				

### Randomization and blinding

Eligible patients were randomly assigned to either the treatment group or the control group in a 1:1 ratio using a computer-generated random number sequence (SPSS version 25.0, IBM Corp., Armonk, NY, USA). The randomization sequence was prepared by an independent statistician who was not involved in patient recruitment or outcome assessment. Allocation concealment was ensured by placing the group assignments in sequentially numbered, opaque, sealed envelopes. The envelopes were opened by a research nurse only after the patient had completed all baseline assessments and was ready for discharge.

Due to the nature of the nursing intervention, it was not possible to blind the patients or the nurses who delivered the intervention. However, outcome assessors [the nurses who performed the activities of daily living (ADL) and Stroke-Specific Quality of Life Scale (SS-QOL) evaluations] and the statistician who performed the data analysis were kept blinded to group allocation. The blinding was maintained by requiring the outcome assessors not to ask about the type of post-discharge care received, and by having the statistician receive only de-identified data with group codes A and B.

**Figure 1:**
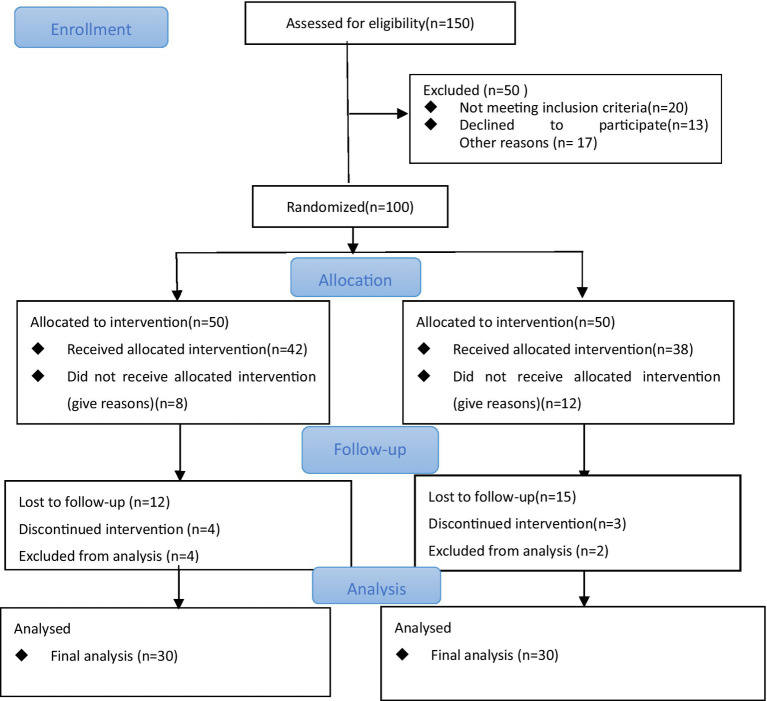
Consolidated Standards of Reporting Trials (CONSORT) flow diagram.

The primary outcome was pre-specified as the change in ADL scores from baseline to 3 months. No formal sample size calculation was performed before the study, as this was an exploratory, hypothesis-generating investigation. The sample size of 30 patients per group was based on practical considerations, including feasibility, study duration, and patient availability at a single center. We acknowledge this as a limitation (see Discussion). To provide a *post-hoc* reference, with 30 patients per group and *α* = 0.05, our study had 80% power to detect an effect size (Cohen’s *d*) of approximately 0.74 for the primary outcome, assuming a two-sample *t*-test (see Figure 1).

## Methods

The principal investigators involved in this study prepared the informed consent form for patients and their families, stating that the study aimed to improve nursing care in the later stage. Whether they chose to participate or not, participants would be treated equally and given the best treatment, so there was no need for concern. Patients participating in the study were clearly informed that they would be divided into experimental and control groups. They were assured that their group assignment would not affect their later recovery. The experts provided professional and patient explanations to the patients and their families. For patients with preserved self-awareness, the study was patiently explained in the form of pictures and texts, and their families were informed. For patients with poor self-awareness, the study was directly explained to their families, and then the patients were included in the study with their consent. Patient records were established for the two groups, including general and medical information and scores of scales such as the ADL and SS-QOL scales. All patients received routine nursing from the Neurology Department during their hospitalization, along with health education guidance on diet, medication, rehabilitation, safety, and basic nursing practices upon discharge.

Patients in the control group received routine continuous nursing from the tertiary hospital after discharge. This consisted of telephone follow-up once every 2 weeks for the first 3 months post-discharge. Each call lasted approximately 10–15 min, during which a trained nurse assessed the patient’s general condition, medication adherence, and any new symptoms. Push notifications of health education materials via WeChat or SMS every week, covering topics such as diet, medication, rehabilitation exercises, and warning signs of stroke recurrence were provided. There were no home visits or community-based interventions. If a patient reported an urgent problem during the telephone call, they were advised to return to the tertiary hospital for assessment.

Patients in the treatment group received 3-month home-based nursing, including weekly home visits initially, tapering to biweekly; phone follow-up every 3 days initially and then weekly; and remote monitoring via WeChat, delivered by community nurses trained by the tertiary hospital.

Standardized intervention protocol and quality control: A written operation manual was developed before the start of the study. Community nurses (*n* = 12) completed a 4-h theoretical and 2-h practical training, requiring a post-training exam score of ≥80% (mean achieved, 89.4%). Each home visit, with the first visit conducted within 7–10 days after discharge, then weekly for month 1 and biweekly for months 2–3; total, approximately eight visits over 3 months, 45–60 min/visit, included six mandatory components: vital signs, medication adherence check, ADL assessment, rehabilitation guidance, complication inspection, including pressure sores, shoulder pain, and signs of pneumonia, and caregiver education. Telephone follow-up was performed every 3 days in month 1 and then weekly (approximately 10 min/call). Remote monitoring used a dedicated WeChat group for a 24-h response. Final outcome assessments were conducted at 3 months after discharge. Fidelity was monitored by random audio recording of 20% of visits/calls, with an adherence rate of 94%. These measures ensured full replicability.

Explicit distinction between intervention and control groups. To eliminate ambiguity and allow assessment of specific intervention effects, the key differences between the two groups are summarized as follows: (1) setting—the treatment group received home-based care delivered by community nurses, whereas the control group received remote care through telephone calls and text messages, without home visits; (2) provider—care in the treatment group was delivered by community nurses trained by the tertiary hospital, whereas care in the control group was delivered directly by tertiary hospital nurses; (3) contact frequency—the treatment group had weekly home visits plus calls every 3 days during the first month, then biweekly visits and weekly calls during months 2–3, whereas the control group had only biweekly telephone calls; (4) content—the treatment group included hands-on rehabilitation guidance, in-person complication inspection, and caregiver teaching, whereas the control group provided only verbal advice and digital educational materials. These clear boundaries ensure that observed improvements could be specifically attributed to the additional community-based, home-delivered components rather than to non-specific factors such as increased attention.

A continuous nursing group for stroke sequelae was established in the medical treatment combination. The group consisted of doctors and nurses from medical institutions at all levels within the medical treatment combination. The members of the group had more than 5 years of working experience and junior professional titles or above. A group leader was appointed with clear responsibilities, and the work of the members was divided so that all members knew their responsibilities. A questionnaire was designed in the group to investigate patients’ understanding of continuous nursing, their acceptance of continuous nursing, the form, content, and frequency of continuous nursing services, the intention, form, and price of charging, their awareness of or willingness to follow the specialized nursing practices in the Neurology Department, and other contents.

Nursing issues related to stroke sequelae were sorted out, and the training program for continuous nursing services for stroke sequelae was established and implemented. Medical specialists and nurses in the Neurology Department established a list of questions for the nursing of stroke sequelae in multiple aspects, including self-care ability, catheter nursing, skin nursing, nursing risks, and other aspects. Questionnaire surveys and theoretical and operational assessments were adopted to understand how well the members of the continuous nursing group from community medical institutions within the medical treatment combination had mastered stroke sequelae continuous nursing knowledge and related specialized neurological nursing skills. The training program for stroke continuous nursing services was specially established according to the question list for stroke nursing. A training schedule was made in detail, and continuous nursing practitioners were provided with theoretical teaching, operational demonstration, clinical guidance, case discussion, and other forms of training. After the continuous nursing service training was completed on schedule, the practitioners were reassessed in theory and operation, and the training effect was evaluated.

Health promotion and education on related knowledge were conducted. Patients and their family members’ poor awareness of the medical treatment combination-based continuous nursing model for stroke sequelae and related medical knowledge may result in psychological resistance. Therefore, the nursing group should assign a person to provide publicity and education to patients and their family members by using models, self-made publicity and educational brochures, knowledge lectures, and other forms before discharge, so that patients and their family members can correctly understand the follow-up rehabilitation of stroke and the stroke sequelae continuous nursing model based on the medical treatment combination, thus enhancing their confidence in treatment and ensuring their active cooperation in the later period. This is of significance for improving the compliance and follow-up rehabilitation of patients (10).

**Figure 2:**
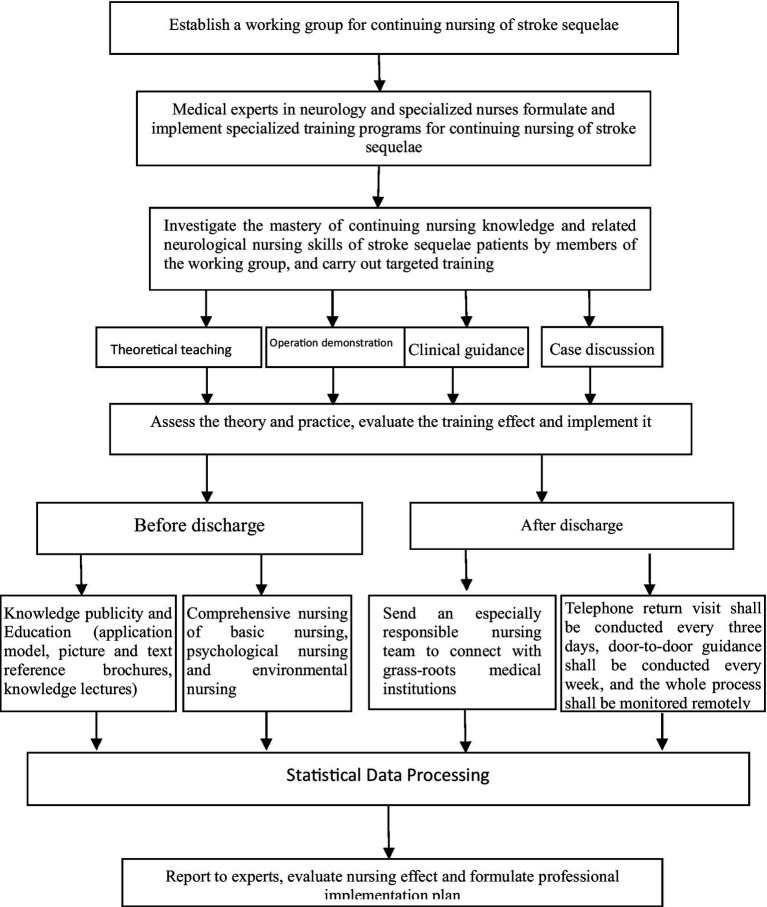
Nursing process.

Patients received home continuous nursing from community medical institutions after discharge. Tertiary hospitals transferred the information of discharged patients to community medical institutions within the medical treatment combination and informed patients and their family members by telephone. Members of the continuous nursing group visited patients in their homes 7–10 days after discharge, and medical workers in tertiary hospitals instructed those in community medical institutions to use multiple scales to evaluate patients’ health status, screen out risk factors for stroke, inquire about and record patients’ home nursing in detail, and instruct patients in proper medication, skin nursing, safety prevention, and other aspects. For patients with catheters, medical workers instructed the patients’ family members to complete catheter nursing according to the nursing routine and evaluated their standardization in completing basic nursing procedures such as nasal feeding, replacement of urine bags, and position changes. A designated person was assigned to conduct a telephone follow-up every 3 days, conduct a professional training every week, and conduct remote monitoring throughout the whole process until the patients recovered more than 80%. For patients with severe symptoms and disability, telephone follow-up was conducted every day and home care every 3 days until their condition improved to mild. After that, the continuous nursing of patients was handed over to medical workers in community medical institutions within the medical treatment combination, and the medical workers provided home continuous nursing for patients on a regular basis. The active intervention period lasted 3 months after discharge. All post-intervention outcome assessments, including ADL, SS-QOL, complications, readmission, medical expenses, and nursing satisfaction, were conducted at the 3-month time point by trained nurses who were blinded to group allocation (see Figure 2).

### Observation indicators

All outcome assessments were performed at 3 months after discharge, that is, at the end of the active 3-month intervention period. The following outcomes were compared between the two groups: ADL score, SS-QOL score, nursing satisfaction, which was assessed by a self-administered questionnaire, probability of complications, defined as the presence of at least one of the following: pressure ulcers, shoulder pain, pneumonia, or urinary tract infection, readmission rate, defined as any unplanned hospitalization for stroke-related reasons within 3 months after discharge, and medical expenses, defined as total direct medical costs during the 3-month post-discharge period, reported in thousands of Chinese yuan (CNY). During the observation period, medical workers in tertiary hospitals monitored patients’ conditions by telephone or WeChat as needed, but the formal outcome assessment was standardized at 3 months.

ADL scale (11): 0–20 points indicate complete dependence; 25–45 points indicate severe dependence; 50–70 points indicate moderate dependence; 75–95 points indicate mild dependence; and 100 points indicate independence.

SS-QOL scale (12): 1 point indicates total difficulty; 2 points indicate great difficulty; 3 points indicate moderate difficulty; 4 points indicate a little difficulty; and 5 points indicate no difficulty.

Follow-up assessments of ADL and SS-QOL were performed at 3 months after discharge, that is, the end of the active intervention period. To rule out measurement bias, all pre- and post-intervention assessments were conducted by two trained nurses who were blinded to group allocation. Interrater reliability was assessed using the intraclass correlation coefficient (ICC) based on 20 randomly selected patients, including 10 per group; the ICC values were 0.92 for ADL and 0.89 for SS-QOL, indicating excellent agreement.

### Statistical analysis

Data from this study were statistically analyzed using SPSS 25.0 statistical software. The enumeration data were described using *n* (%), and the comparison was conducted using the chi-square test; the measurement data were described using mean ± standard deviation, and the comparison was conducted using the *t*-test. With *α* = 0.05 as the test criterion, *p* < 0.05 was considered to indicate a statistically significant difference. All analyses were performed according to the pre-specified statistical plan, with the statistician blinded to group allocation.

## Results

All patients completed the 3-month follow-up period. Outcome data at 3 months after discharge were available for all 60 included patients. All medical expenses are reported in CNY. As shown in Table 3, baseline assessments were performed within 48 h before discharge, and follow-up assessments were performed at 3 months after discharge, that is, the end of the active intervention period. Table 3 lists the nursing effects of the patients in the two groups. The results showed that there was no statistically significant difference in ADL scores, SS-QOL scores, or medical expenses between the two groups before nursing (40.60 ± 11.42 points vs. 38.10 ± 10.34 points, 1.90 ± 0.89 points vs. 2.03 ± 0.93 points, and 740 ± 0.36 CNY vs. 690 ± 0.32 CNY, *p* > 0.05). The ADL and SS-QOL scores in the treatment group were significantly higher than those in the control group after nursing (64.27 ± 12.71 points vs. 49.63 ± 9.82 points and 3.07 ± 1.26 points vs. 2.23 ± 1.07 points, respectively), whereas medical expenses were lower in the treatment group than in the control group (410 ± 0.24 CNY vs. 590 ± 0.20 CNY; *p* = 0.03). Nursing satisfaction in the treatment group was significantly higher than that in the control group (90.0% vs. 63.3%, *p* = 0.015). The probability of complications and the readmission rate in the treatment group were significantly lower than those in the control group (30.00% vs. 63.34 and 16.67% vs. 40.00%, *p* = 0.01), and there was a highly statistically significant difference between the two groups. Specifically, 9 patients (30.0%) in the treatment group experienced at least one complication, compared to 19 patients (63.3%) in the control group (*p* = 0.010). The readmission rate was 5/30 (16.7%) in the treatment group compared to 12/30 (40.0%) in the control group (*p* = 0.045). This indicated that, for patients receiving home continuous nursing from community medical institutions, the ADL and SS-QOL scores were significantly improved, while nursing satisfaction was also significantly improved. In addition, the incidence of complications, the number of readmissions, and the medical expenses decreased significantly. The above data show that after receiving the continuous care mode based on the medical consortium, the patients’ self-care ability and quality of life were significantly improved, and their later-stage recovery was significantly improved. In addition, it also improved the cooperation degree of patients in the later stage of treatment, effectively reduced the repeated admission rate and medical expenses, and reduced the psychological burden and economic pressure of patients. Moreover, compared with the control group receiving routine nursing in tertiary hospitals, each index showed obvious advantages and a remarkable effect.

**Table 3:** Comparison of nursing effects between the two groups.

		Treatment group (*n* = 30)	Control group (*n* = 30)	*χ*^2^/*t* value	*p* value
ADL	Before	40.60 ± 11.42	38.10 ± 10.34	0.889*	0.378^▲^
	After	64.27 ± 12.71	49.63 ± 9.82	4.992*	0.000^▲^
SS-QOL	Before	1.90 ± 0.89	2.03 ± 0.93	−0.570*	0.571^▲^
	After	3.07 ± 1.26	2.23 ± 1.07	2.761*	0.008^▲^
Nursing satisfaction	Before	–	–	5.963*	0.015^▲^
	After	27	19		
Complications	Before	–	–	6.696*	0.010^▲^
	After	9	19		
Readmission^a^	Before	–	–	4.022*	0.045^▲^
	After	5	12		
Medical expenses (CNY)	Before	740 ± 0.36	690 ± 0.32	0.569*	0.571^▲^
	After	410 ± 0.24	590 ± 0.20	−3.12*	0.003^▲^

ADL, activities of daily living; SS-QOL, Stroke-Specific Quality of Life Scale; CNY, Chinese yuan; *χ*^2^, chi-square; −, not applicable.

Before, within 48 h before discharge; after, at 3 months after discharge, that is, the end of the active intervention period.

a Data for individual complications are the number of patients who developed that specific complication; a patient may have more than one complication. The overall complication rate refers to the number of patients with at least one complication.

## Discussion

Stroke is a cerebral blood circulation disorder with the characteristics of acute onset and rapid development. With the aging of society, the incidence of this disease has been increasing year by year, and it is increasingly being observed in younger populations (13, 14). Stroke patients are often accompanied by sequelae such as aphasia and hemiplegia, placing a heavy burden on patients and their families. Later-stage rehabilitation of patients with stroke sequelae is a long-term process, which requires professional and long-term continuous nursing. At present, medical insurance payment systems have improved the service efficiency of hospitals and shortened the average length of stay. An increasing number of patients are discharged after treatment in the acute phase, but they still need professional treatment and nursing (15, 16). However, community medical institutions are incapable of providing high-quality specialized nursing services, especially some specialized technical operations, for these patients (17). Surveys have shown that these patients prefer continuous nursing services from tertiary hospitals to consolidate the effect and promote their rehabilitation. Nevertheless, patients can hardly have access to continuous nursing from tertiary hospitals, which are limited in medical resources. The success of foreign continuous nursing suggests that continuous nursing should be carried out by community medical institutions under the medical treatment combination service system, and its effectiveness is dependent on the community medical service level. The establishment of a medical treatment combination in China creates a new opportunity and platform for the effective development of continuous nursing. At present, many Chinese scholars study and explore the continuous nursing services mainly in specific diseases and the effect of continuous nursing, but few studies have focused on the continuous nursing model based on a medical treatment combination. Therefore, this study discussed the clinical effect of a medical treatment combination-based continuous nursing model on patients with stroke sequelae. The results showed that the nursing satisfaction of the treatment group was higher than that of the control group, and patient compliance was higher (*p* < 0.05). The ADL and SS-QOL scores were significantly higher than those of the control group, while the incidence of complications, the rate of repeated admission, and medical expenses were lower than those of the control group (*p* < 0.05). These data indicated that the continuous nursing model based on medical treatment combination can not only effectively improve the self-care ability and quality of life of patients with stroke sequelae and reduce the burden on their families, but also effectively reduce patients’ complications and readmission rate, and thus their treatment costs. These findings support the clinical effectiveness of this continuous nursing model.

The improved outcomes may be explained by several mechanisms: enhanced medication and rehabilitation adherence through frequent home visits, early detection and management of complications, such as pressure sores and shoulder pain, by trained community nurses, and reduced caregiver burden via tailored education. Compared with the Transitional Care Model (TCM) developed by Bixby and Naylor (6), which focuses on high-risk older adults in the United States, our model specifically addresses the Chinese context, where community healthcare is underdeveloped. Unlike prior Chinese studies that only described the hospital–community linkage concept, our study provides quantified intervention parameters, including training hours, visit frequency, session duration, and fidelity rate, enabling direct replication. This strengthens the evidence base for implementing medical treatment combination-based continuous nursing in resource-limited settings.

In conclusion, in this single-center, exploratory study, a medical treatment combination-based continuous nursing model, that is, home-based care delivered by trained community nurses, significantly improved ADL and SS-QOL scores, reduced complication rates and readmissions, lowered medical costs, and enhanced nursing satisfaction compared with routine telephone-based follow-up in patients with stroke sequelae. These findings suggest that the model holds promise for improving post-discharge outcomes in this population.

We acknowledge several limitations. First, the sample size was relatively small, with 30 patients per group. This limits statistical power and increases the risk of type II errors for secondary outcomes. A formal sample size calculation was not performed prior to the study, as this was an exploratory investigation. *Post-hoc* power analysis indicated that our study had 80% power to detect a large effect, Cohen’s *d* ≈ 0.74, for the primary outcome, but smaller and clinically meaningful effects may have been missed. Second, this was a single-center study conducted at one tertiary hospital in China. The generalizability of our findings to other settings, healthcare systems, or populations with different demographic or cultural characteristics is uncertain. Third, blinding of patients and intervention nurses was not feasible due to the nature of the intervention, that is, home visits vs. telephone follow-up. Although outcome assessors and the statistician were blinded, performance bias cannot be entirely excluded. Fourth, we did not pre-specify a primary outcome or adjust for multiple comparisons, as six outcomes were tested. Therefore, the results for secondary outcomes, including SS-QOL, satisfaction, complications, readmission, and medical costs, should be regarded as exploratory and hypothesis-generating, requiring confirmation in future confirmatory trials. Fifth, we lacked detailed baseline data on stroke severity, such as the National Institutes of Health Stroke Scale, cognitive status, and specific comorbidities, such as hypertension, diabetes, and atrial fibrillation. Although randomization should have balanced these unmeasured confounders, residual confounding cannot be ruled out. Sixth, the follow-up duration was limited to 3 months. Longer-term outcomes, such as 6 or 12 months, remain unknown, and the sustainability of the observed benefits beyond the active intervention period is unclear. Seventh, medical expenses were limited to direct medical costs during the 3-month post-discharge period and did not include indirect costs, such as caregiver time and transportation, or longer-term economic outcomes. We did not use analysis of covariance or repeated-measures analysis to adjust for baseline values, although baseline comparability makes this less critical. Future studies with larger samples should consider these methods. Despite these limitations, the study provides valuable preliminary evidence for the feasibility and potential effectiveness of a medical treatment combination-based continuous nursing model in stroke sequelae care, and it offers a fully replicable protocol for future research.

## Data Availability

The original contributions presented in the study are included in the article/supplementary material, further inquiries can be directed to the corresponding authors.
